# Locomotive Syndrome is a Risk Factor for the Dropout of Continuous Positive Airway Pressure Treatment in Patients with Obstructive Sleep Apnea Syndrome

**DOI:** 10.3390/healthcare8020177

**Published:** 2020-06-19

**Authors:** Hiroaki Kataoka, Nobuyuki Miyatake, Hiromi Mukai, Hirohisa Ichikawa, Yukako Arakawa, Yoshihiro Mori

**Affiliations:** 1 Department of Physical Therapy, Faculty of Health Sciences, Okayama Healthcare Professional University, Okayama 700-0913, Japan; 2 Rehabilitation Center, KKR Takamatsu Hospital, Kagawa 760-0018, Japan; 3 Department of Hygiene, Faculty of Medicine, Kagawa University, Kagawa 760-0018, Japan; miyarin@med.kagawa-u.ac.jp; 4 Sleep and Respiratory Disease Center, KKR Takamatsu Hospital, Kagawa 760-0018, Japan; et_king_dom@yahoo.co.jp (H.M.); ichikawa@kkr-ta-hp.gr.jp (H.I.); arakawa@kkr-ta-hp.gr.jp (Y.A.); mori@kkr-ta-hp.gr.jp (Y.M.)

**Keywords:** obstructive sleep apnea syndrome, locomotive syndrome, continuous positive airway pressure (CPAP) therapy, dropout

## Abstract

*Objective:* The purpose of this study was to investigate the risk factors linked to patient dropout from continuous positive airway pressure (CPAP) therapy for the treatment of obstructive sleep apnea syndrome (OSAS). *Methods:* This study included 1191 patients with OSAS at baseline assessment, who were followed for 3 years. We evaluated clinical parameters, indicators related to OSAS treatment, exercise habits and the presence of locomotive syndrome (LS). LS was evaluated by a ‘loco-check’, as established by the Japanese Orthopedic Association. The OSAS patients were categorized at baseline as belonging to an ‘LS group’ or a ‘non-LS group’, and clinical parameters were compared. *Results:* Eighty-six patients (7.2%) dropped out of CPAP therapy during the 3 year follow-up period. The dropout rate of the LS group was significantly higher than that of the non-LS group. Using a Cox-proportional hazard model, the LS, old age and poor compliance were determined to be significant risk factors for dropping out of CPAP therapy. The hazard ratios (95% CI) of LS, elderly people and poor CPAP compliance were 2.11 (1.31–3.48), 1.80 (1.11–2.94) and 1.61 (1.04–2.47), respectively. *Conclusion:* LS may be the critical risk factor for dropping out of CPAP therapy among patients with OSAS.

## 1. Introduction

Sleep apnea syndrome (SAS) is a typical disease with sleep disorders [1], and is found in 4% of adult men and 2% of women [2]. SAS is classified into either obstructive sleep apnea syndrome (OSAS) or central apnea syndrome, with OSAS representing 90% of SAS cases [3]. OSAS induces apnea due to frequent or partial upper airway obstruction during sleep, alongside gas exchange disturbance and hypoxemia. OSAS patients are two times more likely to have hypertension, two–three times more likely to have ischemic heart disease, and three–five times more likely to have cerebrovascular disease than healthy people [4]. Therefore, OSAS is strongly recognized as a risk factor for lifestyle-related diseases. In addition, it has also been reported to be associated with reduced health-related quality of life (HRQOL) [5].

Since the basic clinical presentation of OSAS is characterized by upper airway obstruction during sleep, maintaining upper airway patency is the main treatment method. Currently, continuous positive airway pressure (CPAP) therapy is the most widely used treatment method. Many previous studies have reported that CPAP therapy improves sympathetic activity [6,7,8], markers of inflammation [9,10], hypertension [11,12], HRQOL [13,14] and left ventricular diastolic function [15]. To ensure these outcomes, continuous treatment with CPAP therapy is very important. On the other hand, CPAP therapy is only applicable to symptomatic patients and cannot completely cure OSAS. Therefore, it is expected that the discontinuation of treatment increases the risk of worsening the condition, alongside various other complications. A previous study [16] investigating the risk factors for CPAP therapy dropout revealed an association with body mass index (BMI). However, some other studies [17,18,19] have reported that BMI does not affect the continuation of CPAP therapy, and there remains no consensus. Although we have previously reported a high prevalence of locomotive syndrome (LS) in OSAS patients (approximately 50%) [20], we are under the impression that OSAS patients with LS are more likely to drop out of CPAP therapy in our clinical practice. Therefore, in this study, we hypothesized that LS may be one of the risk factors for CPAP therapy dropout; to assess this, we evaluate the dropout rates between patients with and without LS using a survival analysis.

## 2. Materials and Methods

### 2.1. Study Design and Subjects

This research was conducted as a cohort study to investigate the risk factors associated with patients with OSAS dropping out of CPAP therapy. The follow-up period was 3 years (initial assessment: from November to December 2016; final assessment: from November to December 2019). This study included 1191 patients with OSAS (1029 males and 162 females) who were treated for CPAP at KKR Takamatsu Hospital, Kagawa, Japan. The apnea hypopnea index (AHI) and respiratory disturbance index (RDI) at OSAS diagnosis were 51.2 ± 25.6 and 53.2 ± 14.2, respectively. Patients were excluded if they had acute or chronic musculoskeletal disorders, other neurological or endocrine disorders, or a history of stroke. Participants provided written informed consent for inclusion before they participated in the study. The research ethics committee of KKR Takamatsu Hospital approved of this cohort study (approval number: E111). The study was conducted in accordance with the Declaration of Helsinki.

### 2.2. Clinical Parameters

We collected data related to age (years), sex, height (cm), body weight (kg), BMI (kg/m^2^), systolic blood pressure (mmHg) and diastolic blood pressure (mmHg), which were measured using standard clinical methods. Treatment periods for OSAS therapy and AHI were assessed based on clinical records. Well-trained medical staff interviewed each patient to evaluate the Japanese version of the Epworth Sleepiness Scale (JESS) [21] and exercise habits (aerobic exercise (e.g., walking or jogging), lasting more than 30 min each time, 3 times weekly, for over half a year). CPAP compliance was defined as ‘good’ if carried out for over 4 h; 70% of the night [22]. The JESS is composed of eight questions, with scores ranging from 0 to 24 points. The cut-off value is 11 points, and the higher the score, the stronger the assessed sleepiness level [23]. 

### 2.3. Evaluation of LS

The loco-check for LS was prepared by the Japanese Orthopedic Association (JOA) in 2007 [24]. According to the JOA proposal, a participant who endorses at least one of the 7 statements on the checklist may have LS. The 7 categories are as follows: (1) you cannot put on a pair of socks while standing on one leg; (2) you stumble or slip in your home; (3) you need to use a handrail when going upstairs; (4) you cannot get across the road at a crossing before the traffic light changes; (5) you have difficulty walking continuously for 15 min; (6) you find it difficult to walk home carrying a shopping bag weighing about 2 kg (e.g., two 1-L milk cartons); and (7) you find it difficult to perform housework requiring physical strength (e.g., use of a vacuum cleaner, moving futons into and out of a closet), according to a previous report [24].

### 2.4. Statistical Analysis

Data were expressed as means ± standard deviation. Subjects were classified as pertaining to the ‘LS group’ or the ‘non-LS group’, according to the results of the loco-check. The Mann–Whitney U-test and χ^2^ test were used to compare the parameters between the LS and non-LS groups at baseline, and a value of *p* < 0.05 was considered to be statistically significant. We obtained Kaplan–Meier curves and used the log-rank test to compare the dropout rate of the LS and non-LS groups. We performed a Cox-proportional hazards regression analysis to evaluate the relationship between the dropout rates and LS. We adjusted for sex, age (<65 or ≥65-years-old), BMI (<25 or ≥25 kg/m^2^), JESS (<11 or ≥11 points), exercise habits, CPAP compliance (poor or good) and treatment period of CPAP therapy (months) in a multivariate model. All data were analyzed using JMP 12.2.0 software (SAS Institute, Cary, NC, USA). 

## 3. Results

The clinical characteristics of the OSAS patients are summarized in Table 1. The mean age, treatment period of OSAS therapy and AHI were 61.4 ± 12.8 years, 69.0 ± 49.0 months and 2.8 ± 3.3 at baseline, respectively. Among the 1191 patients with OSAS, 86 dropped out of CPAP therapy, and three switched to a dental treatment. In addition, there were 16 deaths, and 101 patients (complete therapy: three patients; changing hospital: 98 patients) were lost to follow-up due to retirement. 

We compared the clinical parameters between OSAS patients with and without LS at baseline (Table 2). Five hundred and seventy nine (48.6%) patients had LS. There was a significant difference in sex between the LS and non-LS groups. The age, BMI and treatment period of CPAP therapy of the LS group were significantly higher in OSAS patients with LS than in OSAS patients without LS. The height, body weight and diastolic blood pressure of the non-LS group were significantly higher than those of the LS group. There were 59 (10.2%) and 27 (4.4%) patients in the LS and non-LS group, respectively, who dropped out of CPAP therapy.

**Figure 1:**
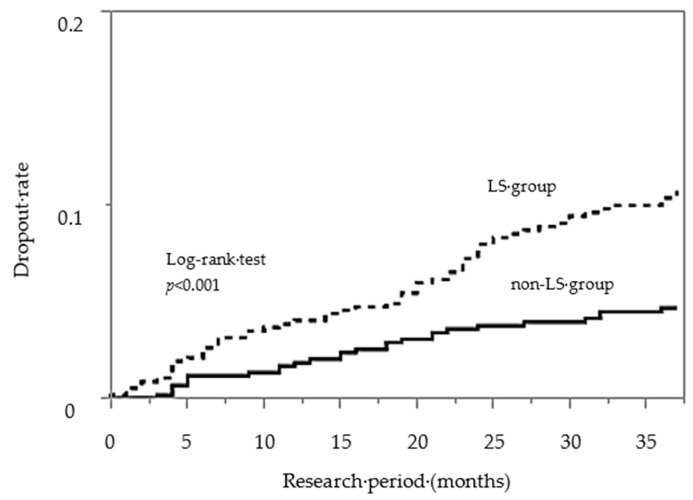
Kaplan–Meier analysis of the rate of dropout of CPAP therapy among OSAS patients.

Figure 1 shows the dropout rate from CPAP therapy, along with the survival analysis. The dropout rate of the LS group was significantly higher than that of the non-LS group (log-rank test *p* < 0.001). The results of the Cox-proportional hazard regression analysis, adjusted for covariates (sex, age, BMI, JESS, exercise habits, LS, CPAP compliance and treatment period of CPAP therapy) are shown Table 3. The data suggested that age, LS and CPAP compliance had a significant effect on dropout from CPAP therapy, even after adjusting for potential confounding effects incurred by the other selected factors considered in the study. In particular, the risk of dropping out of CPAP therapy was higher in the LS than in the non-LS group (HR: 2.11, CI: 1.31–3.48, *p* = 0.002), in elderly patients compared with non-elderly patients (HR: 1.80, CI: 1.11–2.94, *p* = 0.017) and in the poor CPAP compliance compared with good CPAP compliance group (HR: 1.61, CI: 1.04–2.47, *p* = 0.033).

## 4. Discussion

CPAP therapy has been established as a first-line therapy, thanks to its efficacy and safety in the treatment of OSAS [25]. Many studies have reported that continued CPAP therapy reduces mortality [26,27], and that persistence affects life prognosis [28]. Continuing CPAP therapy is very important in the treatment of OSAS, and it is needed for at least 3 years to show demonstrable benefit [16]. Therefore, in this study, OSAS patients treated with CPAP therapy were followed for 3 years, and risk factors for CPAP dropout were examined. Results showed that OSAS patients with LS were more likely to drop out of CPAP therapy. In addition, a Cox proportional hazards analysis showed that LS, old age and poor CPAP compliance were risk factors affecting dropout. Among them, LS was the most strongly related factor.

First, let us consider the finding that LS was a risk factor for dropout. LS is a concept proposed by the JOA in 2007. LS is proposed as a disorder that predominantly affects people who are at a high risk of developing a musculoskeletal ambulation disability attributed to locomotor organ diseases [28]. In LS, the locomotive system consists of three main components: (1) bones, (2) joints and intervertebral discs, and (3) muscles and nerves [29]. LS refers to the conditions under which the elderly have been receiving care services, or high-risk conditions under which they may soon require care services linked to problems in their locomotive organs [30]. LS has been shown to be a risk factor for becoming bedridden by worsening motor impairment [31], and may be closely associated with decreased physical activity and motor capacity. In Japan, approximately 20% of individuals requiring ‘long-term care’ insurance were estimated to have an LS [31]. Therefore, LS is considered to have a high risk of causing a decrease in levels of activity in daily life and quality of life (QOL). In the current Japanese medical system, an OSAS patient undergoing CPAP therapy must undergo a medical examination every three months. Patients may drop out of CPAP therapy because LS makes it difficult for affected patients to visit the hospital. In other words, LS may be indirectly involved, rather than directly involved in dropping out of CPAP therapy. On the other hand, 64.6% of patients in the LS group had exercise habits, which is a higher rate of exercise therapy than that of patients in the non-LS group. Exercise therapy is recommended for OSAS patients as a treatment for preventing and improving LS [32]. Exercise therapy is known to prevent and improve LS, and is recommended for OSAS patients [32]. In this study, exercise habits were evaluated only according to the type of exercise therapy, exercise time and exercise frequency. Therefore, assessment remains subjective, and may not accurately reflect exercise habits. In the future, it will be necessary to evaluate exercise more accurately, using a physical activity meter. In addition, as facilitated by well-trained medical staff such as physical therapists, effective exercise interventions for OSAS patients with LS may reduce the rates of dropout of CPAP therapy. 

Next, let us consider the finding that elderly OSAS patients over 65 years of age and with poor compliance to CPAP therapy were also found to be at risk of dropping out of CPAP therapy. In clinical practice, when interviewing elderly OSAS patients about their use of CPAP therapy, many patients complain of insufficiently understanding the device’s operation and not being able to properly wear the therapeutic mask (mask fitting). In particular, poor mask fitting causes various problems (e.g., air leak and pain). If these problems persist, the usage time and usage frequency of the device may be reduced, resulting in poor CPAP compliance. Several previous studies have reported that OSAS patients have a higher risk of developing dementia than do healthy subjects [33,34]. On the other hand, CPAP therapy has been reported to improve cognitive function [35] and suppress the progression of dementia [36]. In this study, although we were unable to evaluate cognitive function, the cohort was sure to include subjects with lower cognitive function, since the average age of the enrolled patients in this study was 61.4 ± 12.8 years old. Therefore, it is necessary to fully explain to patients how CPAP therapy is being used and how to properly wear a mask when visiting a hospital for a medical examination. 

This study has some limitations. First, in this study, the proportion of male OSAS patients was very high, at 86.4%. Therefore, it remains unclear whether the results of this study can be applied to female OSAS patients. In the future, it will be necessary to assess female SAS patients. Second, this study included patients with mild OSAS (mean AHI: 2.8). The risk factors for dropout in moderate and severe OSAS patients remain unknown. Third, this study only evaluated the risk factors for dropout based on limited medical indicators. Social background (such as family structure and housing environment), comorbidities and psychological aspects may also influence CPAP dropout. Fourth, the type of interface and CPAP pressure may be risk factors for CPAP dropout. However, because of changes in the interface and CPAP pressure during the follow-up period, these effects could not be accurately recorded and proved. Fifth, we also could not investigate the comorbidities of the study population. This factor may also be associated with dropout of CPAP therapy.

## 5. Conclusions

In summary, OSAS patients with LS had a higher rate of dropout of CPAP therapy over the course of the 3 year assessment period. In addition, risk factors associated with CPAP therapy dropout included LS, old age and poor CPAP compliance. Of these, LS was shown to be the most strongly associated factor. The findings from this study may contribute to reducing the rate of CPAP therapy dropout.

## Figures and Tables

**Table 1:** Clinical characteristics of OSAS patients.

Clinical Parameters	Subjects
*n*	1191
Sex (male; n/%)	1029 (86.4)
Age (years)	61.4 ± 12.8
Height (cm)	165.4 ± 8.3
Body weight (kg)	76.0 ± 15.2
BMI (kg/m^2^)	27.7 ± 4.7
Treatment period of CPAP therapy (months)	69.0 ± 49.0
SBP (mmHg)	134.2 ± 11.7
DBP (mmHg)	75.9 ± 10.0
AHI	2.8 ± 3.3
JESS	5.8 ± 4.4
Exercise habits (none; *n*/%)	739 (62.0)
CPAP compliance (poor; *n*/%)	396 (33.2)

Values are presented as means ± SD; OSAS: obstructive sleep apnea syndrome; BMI: body mass index; CPAP: continuous positive airway pressure; SBP: systolic blood pressure; DBP: diastolic blood pressure; AHI: Apnea Hypopnea Index; JESS: Japanese version of Epworth Sleepiness Scale.

**Table 2:** Comparing the clinical parameters of OSAS patients with and without LS at baseline.

Clinical Parameters	LS	Non-LS	*p*
Subjects (*n*/%)	579 (48.6)	612 (51.4)	
Sex			
Males (*n*/%)	461 (44.8)	568 (55.2)	<0.001
Females (*n*/%)	118 (72.8)	44 (27.2)	
Age (years)	66.0 ± 12.1	57.1 ± 11.9	<0.001
Height (cm)	163.4 ± 9.2	167.4 ± 7.0	<0.001
Body weight (kg)	75.3 ± 16.6	76.6 ± 13.6	0.023
BMI (kg/m^2^)	28.1 ± 5.1	27.3 ± 4.2	0.033
Treatment period of CPAP therapy (months)	72.6 ± 49.6	65.5 ± 48.3	0.010
SBP (mmHg)	133.8 ± 12.1	134.5 ± 11.2	0.448
DBP (mmHg)	73.6 ± 10.2	78.0 ± 9.4	<0.001
AHI	3.07 ± 4.01	2.55 ± 2.51	0.209
JESS	5.90 ± 4.49	5.65 ± 4.26	0.778
Exercise habits (none, *n*/%)	374 (64.6)	365 (59.6)	0.078
CPAP compliance (poor, *n*/%)	177 (30.6)	219 (35.8)	0.056

Values are presented as means ± SD; LS: locomotive syndrome; OSAS: obstructive sleep apnea syndrome; BMI: body mass index; CPAP: continuous positive airway pressure; SBP: systolic blood pressure; DBP: diastolic blood pressure; AHI: Apnea Hypopnea Index; JESS: Japanese version of Epworth Sleepiness Scale.

**Table 3:** Cox-proportional hazard regression model depicting the risk of dropping out of CPAP therapy.

Clinical Parameters	HR	95% CI		*p*
Sex (male)	1.17	0.67	2.19	0.588
Age (≥65 years)	1.80	1.11	2.94	0.017
BMI (≥25 kg/m^2^)	1.03	0.65	1.67	0.887
JESS (≥11 points)	0.91	0.44	1.70	0.791
Exercise habits (+)	1.21	0.77	1.87	0.405
LS (+)	2.11	1.31	3.48	0.002
CPAP compliance (poor)	1.61	1.04	2.47	0.033
Treatment period of CPAP therapy	1.00	0.99	1.00	0.782

HR: hazard ratio; BMI: body mass index; JESS: Japanese version of Epworth Sleepiness Scale; LS: locomotive syndrome; CPAP: continuous positive airway pressure; Exercise habits (+): Defined as having exercise habits; LS (+): Defined as having LS.
